# Compatibility between Physical Stimulus Size and Left-right Responses: Small is Left and Large is Right

**DOI:** 10.5334/joc.19

**Published:** 2018-02-27

**Authors:** Peter Wühr, Christian Seegelke

**Affiliations:** 1 Technische Universität Dortmund, DE; 2 Universität Bielefeld, DE

**Keywords:** ATOM, compatibility, congruency, stimulus size, response position, SNARC

## Abstract

According to a theory of magnitude (ATOM, Walsh, 2003, 2015), the cognitive representations of quantity, time, and space share a general magnitude code. Interestingly though, research has largely ignored the relationship between physical (stimulus) size and spatial (response) location. We conducted two experiments investigating compatibility effects between physical stimulus size and left-right responses. In both experiments, right-handed participants responded to a small or a large square stimulus by pressing a left or a right key. In Experiment 1, size was the relevant stimulus feature and we varied the S-R mapping within participants. Results revealed a strong compatibility effect: Performance was better with the compatible mapping (small-left and large-right) than with the incompatible mapping (large-left and small-right). In Experiment 2, participants responded to stimulus color, which varied independently of stimulus size, by pressing a left or right key. Results showed a congruency effect that mirrored the compatibility effect of Experiment 1. The results of our experiments suggest a strong relationship between the cognitive representation of physical (stimulus) size and response location in right-handers. The findings support the notion of a general magnitude code, as proposed in ATOM.

## Introduction

In “*A T*heory *O*f *M*agnitude” (ATOM), Walsh (2003, 2015) proposed a generalized magnitude-processing system in the human brain that uses a common metric for processing information about time, space, number, and other magnitudes. Compelling evidence for a generalized magnitude system has come from neuropsychological and neurophysiological data, suggesting overlapping brain structures for the processing of time, space, and magnitude information in human parietal cortex (e.g., Cohen Kadosh et al., 2007; Kaufmann et al., 2008; see Bueti & Walsh, 2009, for review). According to Walsh (2003, 2015), the magnitude system has evolved in order to support action control, because the successful control of action requires the integration of information about temporal, spatial and quantity-related features of a desired action. ATOM predicts and explains (mutual) interactions and interference effects in the simultaneous processing of information about time, space, number, size, and other magnitudes. With regard to the direction of interference effects, ATOM assumes “some monotonic mapping of quantities: bigger, faster, brighter, further in one domain should correlate with bigger, faster, brighter, further in another” (Bueti & Walsh, 2009, p. 1832). Behavioral studies revealed evidence for relationships between most of the dimensions addressed in ATOM (for reviews, see, Bonato, Zorzi, & Umiltà, 2012; Winter, Matlock, Shaki, & Fischer, 2015), but the relationship between number and space, and the relationship between number and physical size have gained considerably more interest than the relationship between physical size and space. It should be noted that ATOM does not readily predict a particular mapping between magnitudes, such as number or size, and *horizontal* spatial positions. To account for such mapping effects, additional assumptions are required that will be discussed later.

### Number and space

Dehaene, Dupoux and Mehler (1990) first demonstrated a compatibility effect between numerical size and horizontal response location. Their participants classified two-digit numbers as larger than or smaller than a standard, by pressing a left or right key. Results showed that left-hand responses were faster to small as compared to large numbers, whereas right-hand responses were faster to large as compared to small numbers. Dehaene et al. (1993) further explored this so-called “Spatial-Numerical Association of Response Codes” (*SNARC*) effect, and observed some interesting features of the effect. First, the authors demonstrated the SNARC effect in a parity-judgment task, where number magnitude was no longer relevant for the participants’ judgments, indicating that processing number magnitude and activation of the compatible response is automatic to some degree. Second, the authors ruled out handedness as a factor, because left- and right-handed participants showed similar SNARC effects (see, Fischer, 2008, for converging evidence).

To account for the SNARC effect, Dehaene et al. (1993) proposed a spatial representation of number magnitude – the so-called mental number line – that extends from left to right and represents small numbers to the left and large numbers to the right. Based on their observation that the habitual reading and writing direction of the participants affected the direction of the SNARC effect, Dehaene et al. suggested that the mental number line originated as a result of reading and writing habits. Subsequent results on the impact of reading and writing habits on the SNARC effect were inconclusive, however (see Fischer, 2013, for a review). Alternatively, researchers suggested that the mental number line may have originated from counting habits. In particular, studies showed that children prefer to count a row of objects from left to right (e.g., Opfer, Thompson, & Furlong, 2010). Moreover, in finger counting, both children and adults show a preference to start counting on the fingers of their left hand (e.g., Fischer, 2008). Whereas reading and counting habits are ontogenetic sources of a spatial mapping of numbers, the observation of SNARC-like effects in newborn chicks suggest that phylogenetic variables could also play a role (Rugani, Vallortigara, Priftis, & Regolin, 2015). All these sources are not mutually exclusive, however, and it is therefore possible that multiple sources contribute to a spatial mapping of numbers to horizontal locations, as expressed in the SNARC effect (cf. Winter et al., 2015).

### Number and Size

One of the most prominent empirical demonstrations for an interaction between number magnitude und physical size is the “size-congruity effect” (e.g., Henik & Tzelgov, 1982). The size-congruity effect has been demonstrated in number-comparison tasks (e.g., Besner & Coltheart, 1979) and in Stroop-like paradigms (e.g., Tzelgov, Meyer, & Henik, 1992). In a number-comparison task, participants are presented with two numbers varying in numerical and physical size. Besner and Coltheart (1979) showed that judging number magnitude is faster when the irrelevant physical size is congruent rather than incongruent with the to-be-judged numerical magnitude. Henik and Tzelgov (1982) later demonstrated that the size-congruity effect does also occur in the reverse direction: judging physical size is also faster when the irrelevant numerical size is congruent rather than incongruent with to-be-judged physical stimulus size.

The size-congruity effect does also occur in Stroop-like tasks, where participants have to compare either the numerical size or the physical size of a single number stimulus with an internal (i.e., memorized) standard. For example, Tzelgov et al. (1992, Experiments 1 and 2) presented a single digit (2, 3, 4, 6, 7 or 8) in one of two physical sizes at screen center. In the number-judgment task, participants had to indicate whether the digit was numerically larger (or smaller) than the standard 5. In the size-judgment task, participants had to indicate whether the stimulus was physically larger (or smaller) than a standard of intermediate size presented before. A size-congruity effect occurred in both tasks (see, also, Reike & Schwarz, 2017; Santens & Verguts, 2011).

Two general accounts have been proposed for the size-congruity effect (cf. Santens & Verguts, 2011; Schwarz & Heinze, 1998). According to the *shared-representations account*, numerical stimulus size and physical stimulus size are processed in parallel and activate a shared representation at an intermediate level of processing that precedes the decision or response-selection stage. ATOM (Walsh, 2003, 2015) is a prominent example for a shared-representations account. In contrast, according to the *shared-decisions account*, the processing of numerical and physical stimulus size activates independent representations at an intermediate level of processing, but each of these representations can activate a corresponding response code at the subsequent response-selection stage if the response criterion is somehow related to size. Hence, according to the shared-representations account, the size-congruity effect should occur independently of task requirements, whereas the shared-decisions account predicts a size-congruity effect only when the task requires a decision with regard to stimulus size. The shared-decisions account is an instance of so-called dual-route models that have been proposed to explain effects of spatial S-R compatibility (e.g., Kornblum, Hasbroucq, & Osman, 1990; Tagliabue, Zorzi, Umiltà, & Bassignani, 2000; Zorzi & Umiltà, 1995).

The available evidence does not yet allow deciding between the two types of accounts because each has received empirical support. For example, Santens and Verguts (2011) provided evidence for the shared-decisions account by demonstrating that the size-congruity effect depends on task requirements: the effect occurred when the task required a decision with regard to stimulus size (i.e., magnitude judgment), whereas the effect was absent in tasks that required a decision unrelated to size (e.g., parity judgment). More recently, Reike and Schwarz (2017) provided support for the shared-representations account by showing that the congruency between physical and numerical size of a stimulus affects the participants sensitivity for size differences (as measured in d’). According to Reike and Schwarz (2017, p. 387), their results suggest that the processing of numerical and physical stimulus size interact “at an early representational rather than at a late decision stage”.

### Size and space

Although object size is clearly relevant for the control of movements (cf. Jeannerod, 1997), previous research has paid little attention to the relationship between physical size and space. Some recent studies have demonstrated a SNARC-like compatibility effect between the conceptual size of stimuli and response location, extending previous research on non-numerical magnitude (Ren, Nicholls, Ma, & Chen, 2011; Sellaro, Treccani, Job, & Cubelli, 2015; Shaki, Petrusic, & Leth-Steensen, 2012). For example, Ren et al. (Experiment 4), presented participants consecutively two words referring to objects of different size (e.g., apple – mountain). Participants indicated whether the second word denoted an object that was larger or smaller than the first word by pressing a left or right key. Results showed a SNARC-like effect: Left responses were faster to the names of small as compared to large objects, whereas right responses were faster to the names of large as compared to small objects (see, Sellaro et al., 2015; Shaki et al., 2012, for similar findings).

Studies addressing the compatibility between physical stimulus size and horizontal response position are extremely rare: we found only a single published experiment. In this experiment, Ren et al. (2011, Experiment 2) used the same task as in their experiment on conceptual size described above, but used filled circles of different size instead of words as to-be-compared stimuli. The results revealed a statistically significant compatibility effect only for right hand responses (i.e., faster RT for large than for small stimuli), but not for left hand responses. Yet, these results provide suggestive evidence for a connection between the cognitive representations of physical size and space (i.e. positions on the horizontal dimension).

### The present study

In the present study, we wanted to conceptually replicate and extend the research of Ren et al. (2011, Experiment 2). First, in Experiment 1 we aimed at replicating the stimulus size – response location compatibility effect using a classic S-R compatibility task that requires a response (left or right) to a single stimulus in each trial. Second, in both experiments we wanted to test whether the stimulus size – response location effects are restricted to right hand responses (as found in Ren et al., 2011) or can also be obtained in left-hand responses. Third, because size was a relevant stimulus feature in all previous investigations of the stimulus size – response location compatibility effect, we asked in Experiment 2 whether the effect could also be obtained with size as an irrelevant stimulus feature.

## Experiment 1

In Experiment 1 we investigated the relationship between physical stimulus size and horizontal response position with a classic S-R compatibility task. Therefore, stimulus size (small vs. large) was the relevant stimulus feature and participants responded with two S-R mapping conditions in different blocks. In the *compatible* mapping condition, the small stimulus required a left-hand response, whereas the large stimulus required a right-hand response. In the *incompatible* mapping condition, the small stimulus required a right-hand response, and the large stimulus required a left-hand response. Our aim was to conceptually replicate a previous demonstration of the stimulus size – response location compatibility effect by Ren et al. (2011, Experiment 2), and to determine whether the effect would again be more pronounced for the right-hand response.

### Methods

*Participants.* Twenty-four volunteers (20 female, 4 male) with a mean age of 22 years (range 18–27 years) participated in Experiment 1. Participants gave informed consent before the experiment and received course credit for participation. All participants were right-handers (self-report), reported normal or corrected-to-normal visual acuity, and were naive with respect to the purpose of the study.

*Apparatus and stimuli.* Participants sat in front of a 17-inch color monitor, with an unconstrained viewing distance of approximately 50 cm. An IBM compatible computer controlled the presentation of stimuli and recorded the key-press responses. Visual stimuli appeared on a gray background (i.e. E-Prime color “silver”). The fixation point was a small plus sign (Courier font, font size 18). A small square (2 × 2 cm) and a large square (4 × 4 cm) served as imperative stimuli. All stimuli were presented at screen center. Participants responded by pressing the left “Tabulator” key or the right “backspace” key on a standard computer keyboard with QWERTZ layout. The response keys were marked with white adhesive tape.

*Procedure.* At the beginning of the experiment instructions were presented on the monitor describing the task, the S-R mapping, and the sequence of events in a trial. Each experimental trial started with the presentation of a fixation point for 1000 ms. Then the stimulus was displayed until a keypress occurred, or for a maximum period of 2000 ms. A correct response with an RT below 2000 ms was followed by a blank screen for 1500 ms. If a wrong key or no key was pressed within the response period, a corresponding error message was shown for 1500 ms in black color (Courier font, font size 24).

Participants performed a training block (10 trials) and an experimental block with the first S-R mapping followed by a training block (20 trials) and an experimental block with the second S-R mapping. The order of mapping conditions (compatible – incompatible; incompatible – compatible) was counterbalanced across participants. In the compatible mapping condition, the small stimulus required a response with the left (tab) key, whereas the large stimulus required a response with the right (backspace) key. The mapping was reversed in the incompatible mapping condition. Participants operated the left key with the index finger of their left hand and the right key with the index finger of their right hand. Each test block contained 60 trials in random order (2 stimuli × 30 repetitions). Participants could take a rest between blocks, and started the next block at leisure. The experiment took about 15 minutes. The experimenter left the room after the first practice block.

*Data analysis.* Supplementary file 1 contains the raw data from Experiment 1. The data (i.e., individual mean RTs and individual error percentages) were analyzed using separate two-way repeated measures ANOVAs with the factors S-R Mapping (compatible, incompatible) and Response (left, right). The S-R mapping was varied between, the response within blocks.

Trials with RT below 100 ms or above 1,500 ms (less than 1% of trials) were discarded. Partial *eta*^2^ is provided as an effect-size estimate. Because we had a priori expectations concerning the direction of the mapping effect, we conducted one-tailed *t*-tests for examining differences between single conditions.

### Results

**Figure 1:**
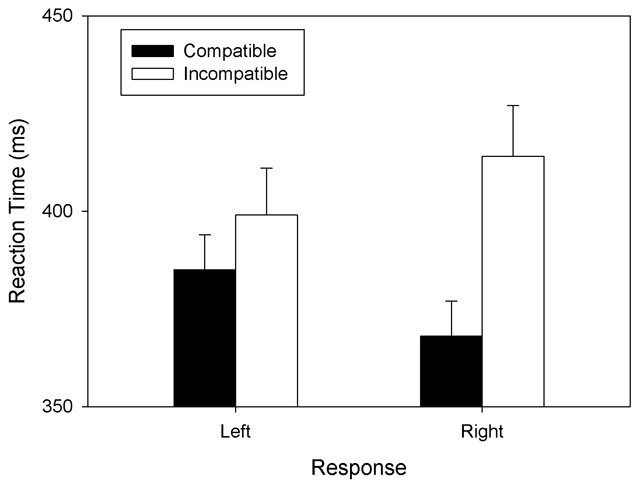
Mean RTs observed in Experiment 1. **Legend:** Mean RTs as a function of S-R Mapping (compatible: small-left; large-right; incompatible: large-left; small-right) and Response (left vs. right) of Experiment 1. Error bars represent standard errors between participants.

Figure 1 shows the mean RTs. A significant main effect of *S-R Mapping* reflected shorter RTs with the compatible mapping (*M* = 377 ms, *SD* = 44) than with the incompatible mapping (*M* = 406 ms, *SD* = 62), *F*(1, 23) = 11.119, *MSE* = 1,923.554, *p* = .003, \eta _p^2 = \,\,.326. A significant S-R *Mapping* × *Response* interaction reflected a numerically larger Mapping effect for the right-hand response (mean difference = 46 ms) than for the left-hand response (mean difference = 14 ms), *F*(1, 23) = 13.621, *MSE* = 424.756, *p* = .001, \eta _p^2\, = \,\,.372. In fact, the mapping effect was significant for the right-hand response, *t*(23) = 4.753, *p* < .001, but only marginally significant for the left-hand response, *t*(23) = 1.401, *p* = .088. The main effect of *Response* was not significant, *F*(1, 23) = 0.013, *MSE* = 712.539, *p* = .911, \eta _p^2\, = \,\,.001.

Table 1 shows the mean error percentages. The ANOVA revealed non-significant main effects of S-R Mapping, *F*(1, 23) = 1.169, *MSE* = 28.618, *p* = .291, \eta _p^2\, = \,\,.048, and Response, *F*(1, 23) = 0.375, *MSE* = 7.724, *p* = .547, \eta _p^2\, = \,\,.016. However, the interaction of S-R Mapping × Response was significant, *F*(1, 23) = 8.886, *MSE* = 9.496, *p* = .007, \eta _p^2 = \,\,.279. In fact, in right-hand responses, there were less errors with the compatible mapping than with the incompatible mapping, *t*(23) = 2.506, *p* = .020, whereas the two mapping conditions did not differ for left-hand responses, *t*(23) = 0.534, *p* = .598.

**Table 1:** Percentages of errors observed in Experiment 1 as a function of S-R Mapping and Response (*N* = 24). Standard deviations are given in parentheses.

	S-R Mapping	
	Compatible	Incompatible

Left Response	3.19 (4.45)	2.50 (3.96)
Right Response	1.67 (2.60)	4.72 (6.44)

### Discussion

In Experiment 1, we conceptually replicated a compatibility effect between physical stimulus size and horizontal response position, which was previously shown by Ren et al. (2011). Specifically, we found that right-hand responses were significantly faster to the larger stimulus than to the smaller stimulus and left-hand responses were (numerically) faster to the smaller stimulus than to the larger stimulus, thus replicating the stimulus size – response location compatibility effect despite several procedural differences between the two studies. In particular, the stimulus set was larger in the former study (including 9 stimulus sizes) than in our experiment (including only 2 stimulus sizes). Moreover, the participants in the former study compared the size of two consecutively presented stimuli in each trial, whereas the participants in our study judged the size of a single stimulus (as being the smaller or larger stimulus in the stimulus set) in each trial. Interestingly, in accordance with the findings of Ren et al. (2011), we also found that the stimulus size – response location compatibility effect was larger for right-hand responses than for left-hand responses.

## Experiment 2

In Experiment 2 we investigated whether the compatibility between stimulus size and horizontal response position would still affect performance when stimulus size was not relevant for the task at hand. Therefore, in the color-discrimination task of Experiment 2, participants responded to stimulus color by pressing a left or right key and stimulus size varied independently from stimulus color. Hence, the task used in Experiment 2 resembles a Simon task with stimulus size instead of stimulus position as the irrelevant feature that is congruent or incongruent with response position (cf. Hommel, 2011, for a review). If congruent conditions (small S – left R; large S – right R) produce better performance than incongruent conditions (large S – left R; small S – right R), we would conclude that stimulus size is involuntarily encoded and automatically primes a congruent response code (cf. Kornblum et al., 1990; Tagliabue et al., 2000; Zorzi & Umiltá, 1995; Gevers, Verguts, Reynvoet, Caessens, & Fias, 2006; for dual-route accounts of congruency effects).

A methodological problem in Experiment 2 concerned the fact that when stimulus size is task-irrelevant, this irrelevant feature may be ambiguous to the participants. In particular, this irrelevant variation may be perceived as a difference in size (of two stimuli presented at the same distance), but it may also be perceived as a difference in distance (of two stimuli with the same size). In order to foster an interpretation in terms of size differences, we introduced a second task where participants had to vocally report the size of the same stimuli also used in the critical color-discrimination task. Half of the participants performed the size-discrimination task before the color-discrimination task; the other half of the participants performed the two tasks in the opposite order. If the irrelevant stimulus feature is in fact ambiguous for participants, the expected congruency effect should be larger for the group that performed the size-discrimination task before the color-discrimination task. If, however, most participants spontaneously interpret the irrelevant variation of the stimulus in terms of size rather than in terms of distance, then both groups should demonstrate a similar stimulus size – response location congruency effect, regardless of task order.

### Methods

*Participants.* Forty volunteers (35 female, 5 male) with a mean age of 24 years (range 19–39 years) participated in Experiment 2. Participants gave informed consent before the experiment and received course credit for participation. All participants were right-handers (self report), reported normal or corrected-to-normal visual acuity, and were naive with respect to the purpose of the study.

*Apparatus and stimuli.* The apparatus was the same as in Experiment 1 with the following exceptions: The orthogonal combination of two sizes (small and large) and two colors (green and red) produced four different stimuli. The stimulus was always presented at screen center. In the size-discrimination task, participants vocally named the size of the stimulus, and the computer measured vocal RT. In the color-discrimination task, participants responded to stimulus color by pressing a left or right key on the keyboard, and the computer registered accuracy and RT of each keypress.

*Procedure.* In Experiment 2, each participant performed two separate tasks. Each task started with the presentation of the instructions on the screen. In the size-discrimination task, participants vocally responded to stimulus size by speaking the German words for “small” or “large” into a microphone. This task involved two blocks of 40 trials (2 stimulus colors × 2 stimulus sizes × 10 repetitions). The experimenter watched participants in the size-discrimination task and observed that errors were extremely rare, but errors were not recorded.

In the color-discrimination task, participants manually responded to stimulus color by pressing a left (tabulator) or right (backspace) key on the keyboard. This task involved a practice block of 20 trials, and two experimental blocks of 60 trials (2 stimulus colors × 2 stimulus sizes × 15 repetitions). In each block, the stimulus displays were presented in random order. The trial structure in both tasks was the same as in Experiment 1. The order of tasks and the S-R mapping in the color-discrimination task (green – left, red – right versus red – left, green – right) were independently counterbalanced across participants.

*Data analysis.* Supplementary file 2 contains the raw data from Experiment 2. The data (i.e., individual mean RTs and individual error percentages) were analyzed using separate mixed-design ANOVAs with the between-subject factor *Task Order* (color discrimination – size discrimination; size discrimination – color discrimination) and the within-subject factors *S-R Congruency* (congruent, incongruent) and *Response* (left vs. right). In congruent conditions, participants made a left response to the color of the small stimulus or a right response to the color of the large stimulus. In incongruent conditions, participants made a left response to the color of the large stimulus or a right response to the color of the small stimulus.

Trials with RT below 100 ms or above 1,500 ms (less than 1% of trials) were discarded. Partial *eta*^2^ is provided as an effect-size estimate.

### Results

**Figure 2:**
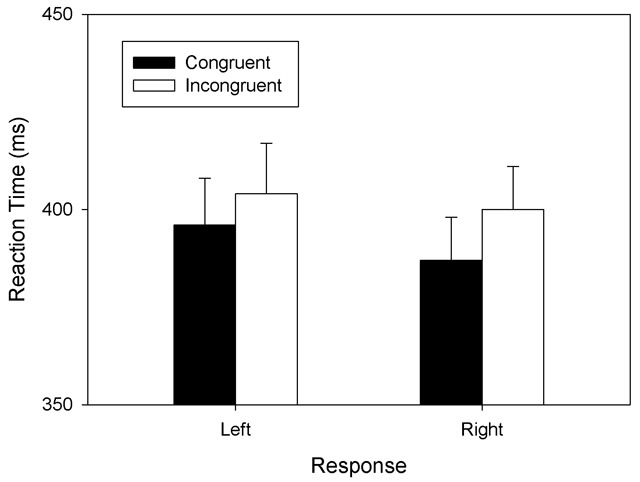
Mean RTs observed in Experiment 2. **Legend:** Mean RTs as a function of S-R Congruency (congruent: small-left; large-right; incongruent: large-left; small-right) and Response (left vs. right) in the color-discrimination task of Experiment 2. Error bars represent standard errors between participants.

A preliminary three-way ANOVA showed that the factor *Task Order* had no significant effect on RTs in the color-discrimination task and, therefore, the variable was excluded from further analyses. A two-way repeated measures ANOVA with *S-R Congruency* and *Response* as independent variables and RTs as dependent variable showed a significant main effect of *S-R Congruency, F*(1, 38) = 21.536, *MSE* = 182.099, *p* < .001, \eta _p^2\, = \,\,.356; RTs were shorter for congruent conditions (*M* = 392 ms, *SD* = 71) than for incongruent conditions (*M* = 402 ms, *SD* = 74). A marginally significant main effect of *Response, F*(1, 38) = 3.650, *MSE* = 459.771, *p* = .063, \eta _p^2\,\, = \,\,.086, reflected a trend towards shorter RTs for right-hand responses (*M* = 394 ms, *SD* = 70) than for left-hand responses (*M* = 400 ms, *SD* = 78). The two-way interaction was not significant *F*(1, 38) = 0.551, *MSE* = 568.361, *p* = .462, \eta _p^2\,\, = \,\,.014. The mean RTs of correct responses are shown in Figure 2.

A three-factorial ANOVA on error-percentages in the color-discrimination task failed to reveal any significant main effect or interaction.

Finally, for the size-discrimination task, a two-way ANOVA on vocal RTs using the within-subject factor Stimulus Size (small, large) and the between-subject factor Task Order revealed a significant main effect of *Stimulus Size*, reflecting shorter RTs to the small stimulus (*M* = 426 ms, *SD* = 62) than to the large stimulus (*M* = 465 ms, *SD* = 78), *F*(1, 38) = 30.936, *MSE* = 973.548, *p* < .001, \eta _p^2\,\, = \,\,.449. This finding may be due to the fact that the voice-key detects the German word “klein” (= small) somewhat earlier than the German word “groß” (= large). The main effect of *Task Order, F*(1, 38) = 0.143; and the two-way interaction, *F*(1, 38) = 2.297, *MSE* = 973.548, *p* = 0.138, \eta _p^2\,\, = \,\,.057, were not significant.

### Discussion

When participants responded to the color of a stimulus, which also varied in size, the relationship between the task-irrelevant stimulus size and response position still produced a congruency effect that mirrored the compatibility effect obtained in Experiment 1. In particular, left-hand responses were faster to the smaller than to the larger stimulus and right-hand responses were faster to the larger than to the smaller stimulus. In accordance with dual-route accounts of the SNARC effect (Gevers et al., 2005) and other congruency effects (e.g., Kornblum et al., 1990; Tagliabue et al., 2000; Zorzi & Umiltá, 1995), this pattern suggests that stimulus size is involuntarily encoded and automatically primes a compatible response code at the response-selection stage.

Two further results of Experiment 2 are noteworthy. First, in contrast to the results of Experiment 1, where the stimulus size – response location compatibility effect was larger in right-hand responses than in left-hand responses, the stimulus size – response location congruency effect was of similar magnitude for the two hands in Experiment 2. We come back to this point in the General Discussion. Second, performing size judgments with the same stimuli before or after the critical color-discrimination task did not affect the stimulus size – response location congruency effect in Experiment 2. Hence, most participants spontaneously perceived the irrelevant variation in stimulus size as intended, and not (so much) as a variation in the distance of two stimuli with similar size.

## General Discussion

Two experiments demonstrated a compatibility effect between physical stimulus size and horizontal response position in right-handed participants. Experiment 1 showed that, when participants responded to the size of a single stimulus, right-hand responses were faster and more accurate to the larger stimulus than to the smaller stimulus, whereas trends in the opposite direction were observed for left-hand responses. This finding replicates a previous finding of Ren et al. (2011) with several methodological modifications and, therefore, demonstrates the reliability of the effect. In Experiment 2 we showed for the first time, that when participants responded to the color of a stimulus varying in size, the compatibility between the now irrelevant stimulus size and response position still affected performance, producing a SNARC- or Simon-like congruency effect. Hence, small stimuli appear to be associated to left responses, whereas large stimuli appear to be associated to right responses. In the following sections, we discuss some theoretical implications of our findings and delineate some directions for future research.

### Theoretical implications

The demonstration of a stimulus size – response location compatibility effect is consistent with ATOM theory (Walsh, 2003, 2015; Bueti & Walsh, 2009). In fact, ATOM assumes a generalized magnitude-processing system (in the brain) where the processing of time, space, number, and other magnitudes overlaps and interacts in order to facilitate the control of complex movements. Therefore, ATOM predicts interference and congruency effects between stimulus size and response position, as demonstrated in our experiments.

However, ATOM does not readily seem to predict the direction of the stimulus size – response location congruency effect observed in our experiments. As mentioned in the introduction, Bueti and Walsh (2009, page 1832) assume some monotonic mapping of quantities: more A should go along with more B. Obviously, however, this prediction can only be applied to describe the relationship between magnitudes with at least ordinal scaling properties, such as size, number or time. The prediction cannot be readily applied to spatial positions such as left or right because horizontal position is a nominal variable. Hence, we would need to explain where the observed relationship between stimulus size and horizontal response position may come from. One possibility is that the mapping between physical size and horizontal location has similar sources as the mapping between numerical size and horizontal location (i.e. the SNARC effect). In that case, the observed mapping of small objects to left and large objects to right would have resulted from several “cultural” variables, such as reading and counting habits, or graphical representations of this mapping (cf. Winter et al., 2015, for examples). Another possibility, which cannot yet be dismissed, is that the mapping between physical size and horizontal location has different sources than the mapping between numbers and horizontal locations. For example, the stimulus size – response location congruency effect might result from functional differences between the two hands. In most people, the right hand is the dominant hand and research has shown that the dominant right hand is stronger than the non-dominant left hand (e.g., Hepping et al., 2015; Incel et al., 2002). Hence, it could be that people have a preference to grasp (and lift) larger objects with their right (and not left) hand because the right hand is stronger than the left hand. Of course, this speculation would require further empirical investigation.

Whereas ATOM is a variant of a “shared-representation account” (cf. Santens & Verguts, 2011), a “shared-decision account” of the stimulus size – response location compatibility effect is also conceivable. For example, dual-route models, which have originally been proposed to explain spatial S-R compatibility effects (e.g., Kornblum et al., 1990; Tagliabue et al., 2000; Zorzi & Umiltà, 1995), could also be applied to the stimulus size – response location compatibility effect. For example, according to Tagliabue et al. (2000), S-R congruency effects between an irrelevant stimulus feature and the response-discriminating feature, as we have observed in Experiment 2, arise from the interaction of short-term and long-term associations between stimulus and response codes, respectively. Short-term associations represent the instructed S-R mapping, that is, the mapping of stimulus colors to left-right response positions in our Experiment 2. Long-term associations, on the other hand, represent some pre-experimentally acquired relationship between irrelevant stimulus size and left-right response positions. When the stimulus is presented, the relevant stimulus feature (i.e., color) activates the correct response code through the short-term association, and the irrelevant stimulus feature (i.e., size) primes the congruent response code through the long-term association. Hence, in congruent conditions, both processing routes activate the correct response, which is quickly selected and executed. In contrast, in incongruent conditions, the long-term association activates an incorrect response that interferes with selection of the correct response, thus increasing RTs and sometimes causing an error. Future research is required to decide empirically between shared-representation or shared-decision accounts of the stimulus size – response location compatibility effect.

### Differences between left and right hands

Interestingly, the stimulus size – response location compatibility was larger for right-hand than for left-hand responses when size was a relevant stimulus feature in Experiment 1, whereas the congruency effect was similar for the two hands when size was an irrelevant feature (Experiment 2). The larger stimulus size – response location compatibility effect for the right hand observed in Experiment 1 replicates similar observations by Ren et al. (2011) in both their Experiment 2 (when physical size was the relevant stimulus) and their Experiment 4 (when conceptual size was the relevant stimulus).

The stronger compatibility effect in right-hand responses cannot be attributed to the different strengths of the associations between small stimuli and left-hand responses, on the one hand, and large stimuli and right-hand responses, on the other hand. If, for example, the association between large and right was stronger than the association between small and left, the stronger association would increase facilitation in congruent conditions with right-hand responses, which is consistent with the findings. However, the stronger association between large and right should also increase interference in incongruent conditions with left-hand responses, which is inconsistent with the findings.

We suggest that the stronger compatibility effect in right-hand as compared to left-hand responses is a mere consequence of a main effect of stimulus size. If the data from Experiment 1 are analyzed as a function of stimulus size (small, large) and S-R compatibility, instead of being analyzed as a function of the response (left, right) and S-R compatibility, you observe significant main effects of stimulus size and S-R compatibility, but no interaction. The main effect of stimulus size reflects shorter RTs to the large stimulus as compared to the small stimulus (e.g., Osaka, 1976), which results from the fact that a larger stimulus is perceptually more salient than a smaller stimulus. Hence, we believe that the two-way interaction between S-R compatibility and response observed in Experiment 1 (and also in Experiment 2 of Ren et al., 2011) is actually the consequence of a main effect of stimulus size on RTs that transforms into a two-way interaction of S-R mapping and response if the data are re-arranged to test for the effects of the two latter variables. In our view, this interpretation is also consistent with the absence of the two-way interaction in Experiment 2 because the main effect of stimulus size should disappear when stimulus size is no longer relevant for selecting a response.

### Conclusion and directions for future research

The results of the present experiments provide evidence for an association between smaller stimulus objects with left-hand responses and between larger stimulus objects with right-hand responses. This association influences response selection regardless of whether stimulus size is relevant or irrelevant for the task at hand, suggesting long-term associations between size and position. Whether these effects arise from overlapping representations of space and magnitude, as suggested by ATOM (Walsh, 2003, 2015), or at the response-selection stage, is a question for future research.

A further question is whether the stimulus size – response location compatibility effect arises with regard to the anatomical status of the (left vs. right) hand or with regard to left or right response positions. This question could be addressed by comparing performance with arms held in parallel, as in the present experiments, to performance with crossed arms. If the effect arises with regard to anatomical hand status, then the participant’s handedness may modulate the effect as well. Hence, it might be interesting to compare the results from right-handed participants, which were addressed in the present experiments, to the results from left-handed participants.

## Data Accessibility Statement

The raw data from both experiments have been published as additional (supplementary) files for this article (see below).

## Additional Files

The Additional files for this article can be found as follows:

10.5334/joc.19.s1 Supplementary file 1. Raw data from Experiment 1 (Wuehr_Seegelke_Raw_Data_Experiment1.csv).

10.5334/joc.19.s2 Supplementary file 2. Raw data from Experiment 2 (Wuehr_Seegelke_Raw_Data_Experiment2.csv).
